# Association of Behçet disease with psoriasis and psoriatic arthritis

**DOI:** 10.1038/s41598-021-81972-4

**Published:** 2021-01-28

**Authors:** Hyung Jin Hahn, Sang Gyu Kwak, Dong-Kyu Kim, Jong-Yeup Kim

**Affiliations:** 1 Department of Dermatology, College of Medicine, International St. Mary’s Hospital, Catholic Kwandong University, Incheon, Republic of Korea; 2 Department of Medical Statistics, School of Medicine, Daegu Catholic University, 33, Duryugongwon-ro 17-gil, Nam-gu, Daegu, Republic of Korea; 3 Department of Otorhinolaryngology-Head and Neck Surgery, Chuncheon Sacred Heart Hospital, Hallym University College of Medicine, 77 Sakju-ro, Gangwon-do, Chuncheon, 24253 Republic of Korea; 4 Institute of New Frontier Research, Hallym University College of Medicine, Chuncheon, Republic of Korea; 5 Department of Otorhinolaryngology-Head and Neck Surgery, College of Medicine, Konyang University, Daejeon, Republic of Korea; 6 Department of Biomedical Informatics, College of Medicine, Konyang University, 158 Gwanjeodong‐ro, Seo‐gu, Daejeon, 35365 Republic of Korea; 7 Myunggok Medical Research Institutue, College of Mediine, Konyang University, Daejeon, 35365 Republic of Korea

**Keywords:** Cellular immunity, Chronic inflammation

## Abstract

Behçet disease (BD) is a debilitating multi-systemic vasculitis with a litany of muco-cutaneous manifestations and potentially lethal complications. Meanwhile, psoriasis (PSO) is a cutaneous and systemic inflammatory disorder marked by hyperplastic epidermis and silvery scales, which may be accompanied by a distinct form of arthropathy called psoriatic arthritis (PsA). While the clinical pictures of these two are quite different, they feature some important similarities, most of which may stem from the autoinflammatory components of BD and PSO. Therefore, the aim of this study was to investigate the prospective link between BD and cutaneous and articular manifestations of psoriasis. BD, PSO, and PsA cohorts were extracted using the National Health Insurance Service of Korea database. Using *χ*^2^ tests, prevalence of PSO and PsA with respect to BD status was analysed. Relative to non-BD individuals, those with personal history of BD were nearly three times more likely to be diagnosed with PSO. The adjusted odds ratio (aOR) was 2.36 [95% confidence interval (CI), 1.91–2.93, *p* < 0.001]. Elevated PSO risk was more pronounced in the male BD cohort (aOR = 1.19, 95% CI 1.16–1.23, *p* < 0.001). In age-group sub-analysis, individuals over 65 years with PSO were one and a half times more likely to be affected with BD, relative to those under 65. The adjusted OR for the older group was 1.51 (95% CI 1.43–1.59, *p* < 0.001). BD individuals with “healthy” body weight were significantly *less* likely to be affected by PSO (aOR = 0.59, 95% CI 0.57–0.62, *p* < 0.001). On the other hand, there was a correlation between BMI and the risk of BD, with the “moderately obese (30–35 kg/m^2^)” group having an aOR of 1.24 (95% CI 1.12–1.38, *p* < 0.001). BD patients were also twice more likely to be associated with PsA (aOR = 2.19, 95% CI 1.42–3.38, *p* < 0.001). However, in contrast to the case of psoriatic disease itself, females were exposed to a greater risk of developing BD compared to the male PsA cohort (aOR = 2.02, 95% CI 1.88–2.16, *p* < 0.001). As with PSO, older BD patients were exposed to a significantly higher risk of developing PsA (aOR = 3.13, 95% CI 2.90–3.40, *p* < 0.001). Behçet disease may place an individual at a significantly increased risk of psoriasis, and still greater hazard of being affected with psoriatic arthritis. This added risk was pronounced in the male cohort, and tended to impact senile population, and this phenomenon may be related with the relatively poor prognosis of BD in males and PSO in older patients.

## Introduction

Behçet disease (BD), a prototype that heads a group of unique inflammatory disorders labelled “variable-vessel systemic vasculitides”, presents a daunting challenge not only in the field of dermatology but across multiple other disciplines in medical science. The earliest written evidence of the illness can be probed back to the time of Hippocrates^1^. Some two millennia later, Benediktos Adamantiades hinted at the possibility that the ocular, muco-cutaneous, and joint manifestations may all be related to one another^2^. Then, it was Hulusi Behçet who formally reported the constellation of these perplexing signs as a discrete and syndromic entity in 1937.

The spectrum and severity of disease presentation can be as diversifying as any multi-systemic vascular disorder, from self-limited mucocutaneous findings, to debilitating ophthalmologic manifestations, and invasion into large-sized vessels and the nervous system, some of which cases can be fatal^3^.

While persons of any race can fall victim to BD, it apparently does not affect all ethnic groups equally. Dubbed “Silk Road Disease” by the locals, the highest incidence of BD has typically been seen in the Eastern Mediterranean Basin and the Middle East, with the figures ranging between 20 and 400 persons per 100,000 population^4,5^. Within the Western Hemisphere BD remains relatively obscure in terms of awareness among the lay public, presumably due to the relatively low incidence^6^ (less than 1 case per 100,000). However, in recent years there has been a surge of renewed interests on the enigmatic condition within the academic communities of Europe and the United States. The phenomenon may be attributed to the attraction BD holds as a *pan-systemic* disease, and the relatively well-founded genetic basis. The latter is perhaps epitomised by the evidence of the strong link to HLA-B51 allele, and the discovery of several other potential genetic loci, which was facilitated by such exhaustive analytic tools as whole genome association study^7,8^ (WGAS).

BD is an idiopathic disease with many contributory factors, such as infection, genetics, and external stimuli, being implicated as culprits^9–11^. Presently BD is loosely categorized as an *autoinflammatory* disorder, but autoimmune mechanisms, via various cytokines, also seem to contribute significantly^12–14^. In that regard, many intriguing parallels can be drawn between BD and psoriasis (PSO), which is another chronic, relapsing inflammatory skin disorder often accompanied by involvement of joints (psoriatic arthritis or PsA), along with a litany of systemic manifestations (Table 1).

**Table 1:** Comorbidities that are most frequently shared by BD and PSO. OR, odds ratio (95% confidence intervals); BD, Behçet disease; PSO, psoriasis.

System	Comorbidity	OR	
		BD	PSO
Musculoskeletal	Rheumatoid arthritis	5.33 (2.45, 12.66)	1.20 (1.01,1.42)
Cardiovascular	Deep venous thrombosis	4.80 (2.42, 9.54)	1.21 (1.16, 1.26)
	Pulmonary embolism	4.64 (2.66, 8.09)	1.46 (1.29, 1.66)
	Myocardial infarction	3.09 (1.28, 7.44)	1.78 (1.51, 2.11)
Gastrointestinal	Inflammatory bowel disease	2.53 (1.92, 3.34)	1.70 (1.50, 2.00)
Psychiatric	Depression	3.87 (1.07, 13.94)	1.39 (1.37, 1.41)
	Anxiety disorder	3.21 (1.01, 10.24)	1.31 (1.29, 1.34)
Metabolic	Hypertension	4.20 (1.50, 11.40)	1.58 (1.42, 1.76)
	Diabetes	4.23 (2.08, 8.66)	1.27 (1.16, 1.40)
	Dyslipidemia	2.67 (1.10, 6.70)	1.88 (1.22, 2.91)

Although the possibility of certain etiological link between these immune-mediated skin conditions has been entertained for some time now, the question so far has not been adequately addressed, at least on a population level. Taking advantage of the large, population-based cohort data from the National Health Insurance Service of Korea (NHIS) database, the authors decided to delve further into the issue in hope of gaining some valuable insights into the pathogenesis of the two unique entities of dysregulated cutaneous immunity.

## Results

### Basic demographics

The study cohort was drawn from a pool of 1,113,656 registered subjects on the DB, with nearly equal sex distribution. Geographically, the highest proportion of the cohort was drawn from Seoul and *Gyeonggi* Province, a metropolitan area surrounding the capital city. Other major cities and their metropolitan provinces of the country were also evenly represented. The cohort was divided into ten income brackets (deciles), and then regrouped as *lower* (brackets 1 through 4), *middle* (brackets 5 through 7), or *upper* (brackets 8 through 10) income tiers. The study cohort was also divided from grade of 0 to 6 according to the extent of their disability, if present (Table 2).

**Table 2:** Baseline characteristics. BD, Behçet disease; PSO, psoriasis; PsA, psoriatic arthritis.

Total no. of patients	*n*	%
	1,113,656	100.0
**Sex**		
Male	558,186	50.12
Female	555,470	49.88
**Age (years)**		
0	108,614	9.75
1–4	52,082	4.68
5–9	74,637	6.70
10–14	70,962	6.37
15–19	70,135	6.30
20–24	84,128	7.55
25–29	85,303	7.66
30–34	97,177	8.73
35–39	88,505	7.95
40–44	93,776	8.42
45–49	72,811	6.54
50–54	53,333	4.79
55–59	43,015	3.86
60–64	42,753	3.84
65–69	31,891	2.86
70–74	20,939	1.88
75–79	12,850	1.15
80–84	7063	0.63
85+	3682	0.33
**Age group**		
≤ 65	1,037,231	93.14
> 65	76,425	6.86
**BMI (kg/m**^**2**^**)**		
< 18.5	547,378	49.15
18.5 ≦ BMI < 23	248,102	22.28
23 ≦ BMI < 25	138,010	12.39
25 ≦ BMI < 30	159,527	14.32
30 ≦ BMI < 35	18,638	1.67
≧ 35	2001	0.18
**Municipalities/provinces**		
Seoul	234,672	21.07
Busan	84,425	7.58
Daegu	57,746	5.19
Incheon	59,424	5.34
Gwangju	32,869	2.95
Daejeon	33,364	3.00
Ulsan	25,447	2.28
Sejong	60	0.01
Gyeonggi-do	234,762	21.08
Gangwon-do	33,919	3.05
Chungcheongbuk-do	34,446	3.09
South Chungcheong	44,401	3.99
Jeollabuk-do	44,207	3.97
Jeollanam-do	46,423	4.17
Gyeongsangbuk-do	62,631	5.62
Gyeongsangnam-do	72,089	6.47
Jeju	12,771	1.15
**Income deciles**		
0	28,332	2.54
1	63,625	5.71
2	65,585	5.89
3	77,713	6.98
4	91,386	8.21
5	105,376	9.46
6	119,392	10.72
7	131,489	11.81
8	142,484	12.79
9	145,778	13.09
10	142,496	12.80
**Income brackets**		
0–4 (lower)	326,641	29.33
5–7 (middle)	356,257	31.99
8–10 (high)	430,758	38.68
**Disability**		
No disabilities	1,087,242	97.63
Moderate (grade 1–2)	8943	0.80
Severe (grade 3–6)	17,471	1.57
**BD**		
No	1,111,426	99.80
Yes	2230	0.20
**PSO**		
No	1,111,489	98.44
Yes	17,362	1.56
**PsA**		
No	1,109,992	99.67
Yes	3664	0.33

### Psoriasis (PSO)

#### Demographic variables

Table 3 shows the prevalence of PSO according to BD status. In comparison to those with no personal history of psoriatic disease, PSO patients were more than twice likely to be diagnosed with BD (Table 4). The adjusted odds ratio(aOR), accommodating sex, age, BMI (body mass index), and income bracket, was 2.36 (95% CI 1.91–2.93, *p* < 0.001).

**Table 3:** Prevalence of psoriasis by Behçet disease status. BD, Behçet disease; PSO, psoriasis.

	PSO, *n*(%)		*p* value
	No	Yes	
**BD**			
No	1,094,151 (99.80)	17,275 (99.50)	< 0.001*
Yes	2143 (0.20)	83 (0.50)	

*Statistically significant for *p* < 0.05

**Table 4:** Prevalence of psoriasis by Behçet disease status, with subgroup analyses by sex, age, and income levels. BD, Behçet disease; PSO, psoriasis; OR, odds ratio; CI, confidence interval (95% confidence intervals).

PSO	Crude			Adjusted^a^			Adjusted^b^		
	OR	95% CI	*p* value	OR	95% CI	*p* value	OR	95% CI	*p *value
**BD**									
No	1	–	–	1	–	–	1	–	–
Yes	2.57	2.07, 3.19	< 0.001*	2.37	1.91, 2.94	< 0.001*	2.36	1.91, 2.93	< 0.001*
**Sex**									
Male				1	–	–	1	–	–
Female				0.84	0.81, 0.86	< 0.001*	0.84	0.81, 0.86	< 0.001*
**Age**									
≤ 65				1	–	–	1	–	–
> 65				1.51	1.44, 1.59	< 0.001*	1.51	1.43, 1.59	< 0.001*
**BMI**									
< 18.5				1	–	–	1	–	–
18.5–23				0.59	0.57, 0.62	< 0.001*	0.59	0.57, 0.62	< 0.001*
23–25				1.14	1.09, 1.20	< 0.001*	1.14	1.09, 1.19	< 0.001*
25–30				1.16	1.11, 1.21	< 0.001*	1.16	1.11, 1.21	< 0.001*
30–35				1.24	1.12, 1.37	< 0.001*	1.24	1.12, 1.38	< 0.001*
≧ 35				1.00	0.72, 1.39	0.98	1.00	0.72, 1.40	0.960
**Income decile**									
0–4							1	–	–
5–7							0.99	0.95, 1.03	0.032
8–10							1.05	1.01, 1.09	< 0.001*

^a^Adjusted result by sex (male and female) and age (≤ 65 and > 65).

^b^Adjusted result by sex (male and female), age (≤ 65 and  > 65) and income(0–4, 5–7 and 8–10).

*Statistically significant for *p* < 0.05.

In terms of gender consideration, this pattern of added BD risk was more pronounced in the male PSO cohort compared to the female counterpart. The aOR for females with respect to males was 1.19 (95% CI 1.16–1.23, *p* < 0.001).

For age group sub-analysis, the study cohort was dichotomized into younger and older groups using 65 years as a cut-off point. The analysis led to the conclusion that older individuals with PSO are one and a half times more likely to be affected with BD, in comparison to the younger (< 65 years) group. The aOR for the former was calculated to be 1.51 (95% CI 1.43–1.59, *p* < 0.001).

#### Body mass index (BMI)

To assess the influence of obesity on the PSO-BD relationship, the study cohort was stratified according to BMI value. With respect to the underweight group (BMI < 18.5 kg/m^2^), BD individuals with “healthy” body weight were significantly *less* likely to be affected by PSO (aOR = 0.59, 95% CI 0.57–0.62, *p* < 0.001). On the other hand, there was a correlation between BMI and the risk of BD, with the “moderately obese (30–35 kg/m^2^)” group having an aOR of 1.24 (95% CI 1.12–1.38, *p* < 0.001). The result for individuals with even greater degree of obesity was not statistically significant, due to an inadequate sample size.

#### Socioeconomic status (SES)

We also performed a sub-analysis based on gross income level of the subjects. The effect of PSO on BD was shown to be consistent across the subgroups. With the lower tier (income brackets 0 through 4) taken as a reference, the middle tier showed an OR of 0.98 (95% CI 0.95–1.03, *p* = 0.032). Meanwhile, the value for the higher tier was 1.05 (95% CI 1.01–1.09, *p* < 0.001).

#### Disability

In those without any significant impairment (Grade “0”), the risk of BD was shown to increase by a factor of 2.5 (OR = 2.55, 95% CI 2.33–2.77, *p* < 0.001). For individuals with mild disability (Grade 1 through 2), the OR was 2.97 (95% CI 2.13–3.82, *p* = 0.028). However, the association was not statistically significant for the “severe (Grade 3 through 6)” subgroup (OR = 2.66, 95% CI 0.70–4.62, *p* = 0.322).

### Psoriatic arthritis (PsA)

#### Demographic variables

Table 5 shows the prevalence of PsA according to BD status. Roughly one-fifth of the PSO cohort had PsA concomitantly. Similar to psoriatic skin disease, patients with a personal history of the inflammatory arthritis were roughly twice more likely to be affected by BD (aOR = 2.19, 95% CI 1.42–3.38, *p* < 0.001, Table 6).

**Table 5:** Prevalence of psoriasis by Behçet disease status. BD, Behçet disease; PsA, psoriatic arthritis.

	PsA, *n* (%)		*p *value
	No	Yes	
**BD**			
No	1,107,783 (99.80)	3643 (99.43)	< 0.001*
Yes	2209 (0.20)	21 (0.57)	

*Statistically significant for *p* < 0.05.

**Table 6:** Prevalence of psoriatic arthritis by Behçet disease status, with subgroup analyses by sex, age, and income levels. BD, Behçet disease; PsA, psoriatic arthritis; OR, odds ratio (95% confidence intervals); CI, confidence interval.

Variable	Crude			Adjusted^a^			Adjusted^b^		
	OR	95% CI	*p* value	OR	95% CI	*p* value	OR	95% CI	*p* value
**BD**									
No	1	–	–	1	–	–	1	–	–
Yes	2.89	1.88, 4.45	< 0.001*	2.20	1.42, 3.38	< 0.001*	2.19	1.42, 3.38	< 0.001*
**Sex**									
Male				1	–	–	1	–	–
Female				2.02	1.88, 2.16	< 0.001*	2.02	1.88, 2.16	< 0.001*
**Age**									
≤ 65				1	–	–	1	–	–
> 65				3.15	2.90, 3.41	< 0.001*	3.13	2.89, 3.40	< 0.001*
**BMI**									
< 18.5				1	–	–	1	–	–
18.5–23				0.34	0.31, 0.38	< 0.001*	0.34	0.31, 0.38	< 0.001*
23–25				1.39	1.27, 1.53	< 0.001*	1.39	1.27, 1.53	< 0.001*
25–30				1.63	1.49, 1.78	< 0.001*	1.63	1.49, 1.77	< 0.001*
30–35				1.86	1.56, 2.23	< 0.001*	1.86	1.56, 2.23	< 0.001*
≧35				1.24	0.66, 2.31	0.667	1.24	0.66, 2.31	0.664
**Income decile**									
0–4							1	–	–
5–7							0.97	0.89, 1.05	0.265
8–10							1.02	0.94, 1.10	0.302

^a^Adjusted result by sex (male and female) and age (≤ 65 and > 65).

^b^Adjusted result by sex (male and female), age (≤ 65 and > 65) and income (0–4, 5–7 and 8–10).

*Statistically significant for *p* < 0.05.

However, in contrast to PSO, PsA females were exposed to a greater risk of developing BD compared to the male PsA cohort (aOR = 2.02, 95% CI 1.88–2.16, *p* < 0.001). Meanwhile, senility was found to increase the risk of BD more dramatically compared to the case of PSO. The aOR for those aged over 65 years was 3.13 (95% CI 2.90–3.40, *p* < 0.001).

#### Body mass index (BMI)

There was a similar relationship between BMI and BD risk in PsA patients. BD patients with optimal body weight were almost three times less likely to develop PSO (aOR = 0.34, 95% CI 0.31–0.39, *p* < 0.001). The aOR for the “moderately obese” was nearly two (aOR = 1.86, 95% CI 1.56–2.23, *p* < 0.001). The result for patients with a BMI of over 35 kg/m^2^ was again inconclusive due to a small sample size.

## Discussion

On the surface, BD and PSO render drastically different pictures. The former most often presents with painless oral or genital ulcers accompanied by nonspecific cutaneous lesions, such as those of erythema nodosum^15^ (EM), while with the latter is accentuated by erythematous and nonpruritic scaly plaques on exposed skin surfaces^16,17^. Aside from the conspicuous clinical features, however, these two share key common ingredients that play a major role in the immuno-pathophysiology, namely deranged Th1/Th17 regulation^18^, and the involvement of autoinflammation^19–21^. The word *autoinflammation* was coined only in recent years, and there are ongoing efforts to cement its conceptual framework. At its inception the term was primarily used to denote a singular group of pyretic conditions of monogenic origin, collectively referred to as periodic fever syndromes. Many of the single-gene abnormalities in these autoinflammation syndromes involve genetic loci that code for vital components in proinflammatory cytokine/chemokine pathways or their associated receptors^22,23^, perhaps the best-known example being *MEFV* in Familial Mediterranean Fever^24^ (FMF). Since then the concept was gradually expanded to encompass a handful of polygenic inflammatory disorders as well, including BD and more recently PSO, entities in which the traditional concept of autoimmunity could not by itself fully account for the pathophysiology.

**Figure 1:**
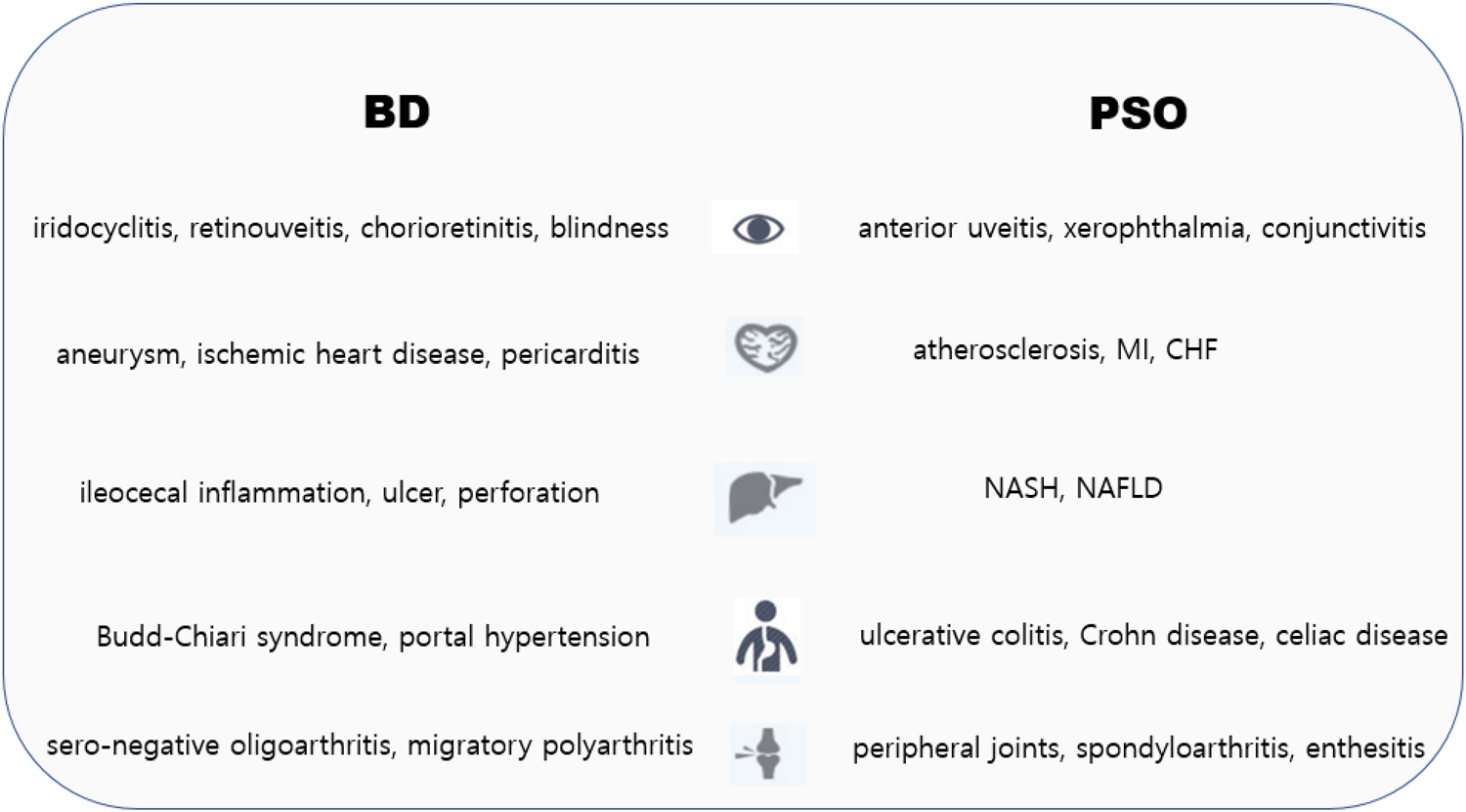
Extracutaneous manifestations of BD and PSO. PSO, psoriasis; BD, Behçet disease; MI, myocardial infarction; CHF, congestive heart failure; NASH, non-alcoholic steatohepatitis; NAFLD, non-alcoholic fatty liver disease.

**Figure 2:**
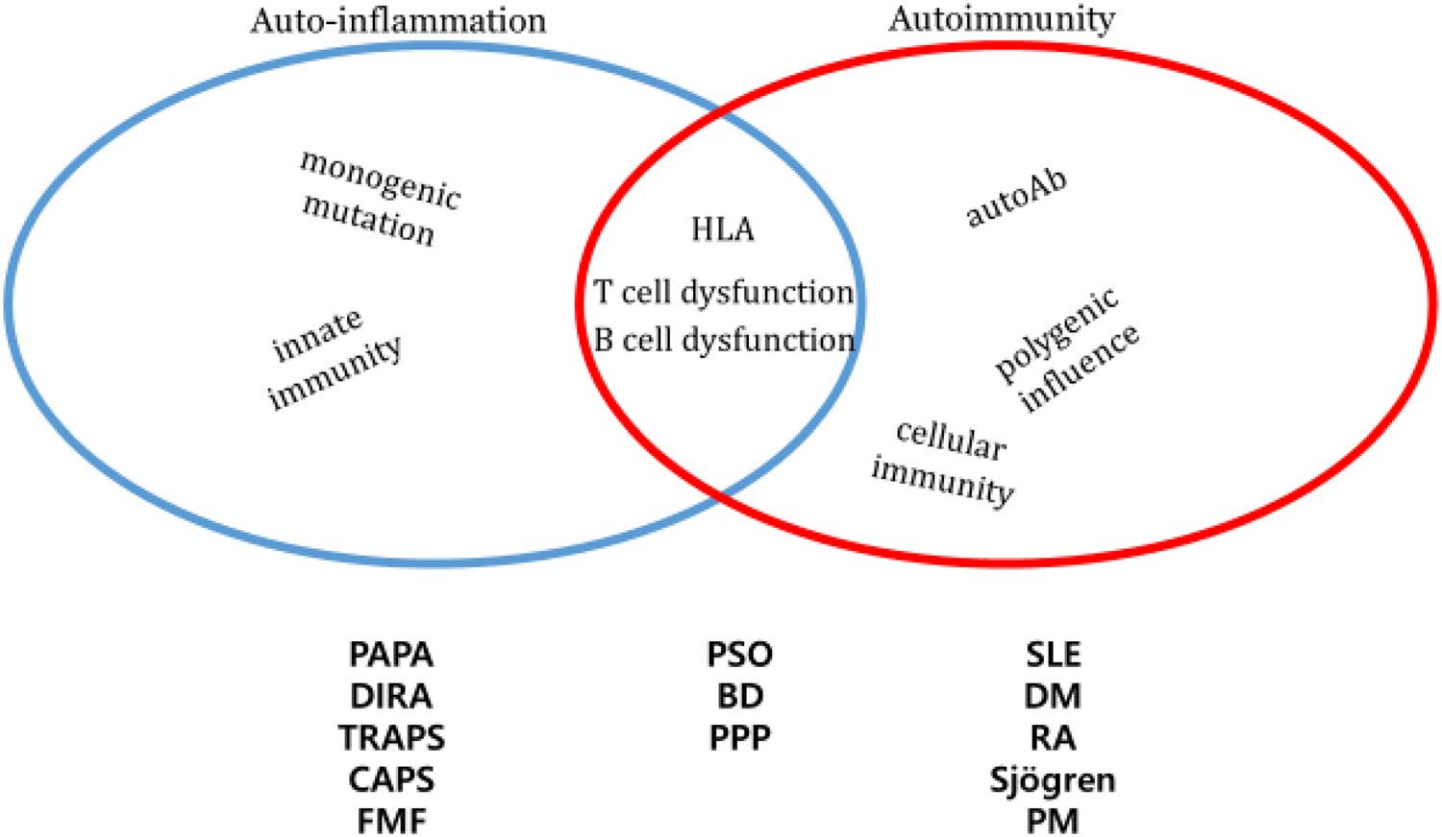
Features of autoinflammation and autoimmunity. HLA, human leukocyte antigen; Ab, antibody; PSO, psoriasis; PPP, pustulosis palmaris et plantaris; BD, Behçet disease; PAPA, pyogenic arthritis, pyoderma gangrenosum and acne syndrome; DIRA, deficiency of the interleukin 1 (IL-1) receptor antagonist; CAPS, cryopyrin-associated autoinflammatory syndromes; FMF, Familial Mediterranean fever; SLE, systemic lupus erythematosus; DM, diabetes mellitus; RA, rheumatoid arthritis; PM, polymyositis.

BD and PSO are something of outcasts in the realm of what are commonly understood as “autoimmune disorders”, for they exhibit pathological features pertinent to both autoimmunity and auto-inflammation^25,26^. In addition, BD and PSO are characterized by similar extracutaneous manifestations (Fig. 1). Clinically, this peculiarity translates into partial or complete absence of several laboratory and histopathological traits that are considered hallmarks of autoimmunity, such as detectable serum auto-antibodies (e.g., anti-DNA or anti-nuclear), female preponderance, and the predominance of lymphocytes as the primary effectors of antigen-independent immune inflammation^27,28^ (relative to neutrophils and monocytes). On the other hand, the autoinflammatory disorders still reserve a number of key cellular and molecular characteristics that are typically associated with autoimmune tendencies, including dysregulation of both cellular and humoral immunity^29^, and the strong ties to specific HLA (human leukocyte antigen) alleles^30^ (Fig. 2). Another distinct attribute that is often used to draw a line between the two is the starring role of innate immunity in the attack directed against host tissues^31^. Presently, however, whether autoinflammation and autoimmunity are mutually exclusive, or merely divergent points on a spectrum of abnormal conditions in which self-tolerance is disrupted, is a matter of considerable debate.

Despite these shared features in immune-pathophysiology, how BD and PSO/PsA may interact with each other in terms of disease behaviour is not well understood. Up to this point, the hypothesis has not been tested seriously with large-scale data, although scattered case reports or patient series have consistently pointed out that the former may place the affected individuals at an augmented risk of the latter^32^, or somehow act as a precipitating factor. Against this backdrop, the present cohort study has estimated that PSO patients are about 2.5 times more likely to be affected with BD. On further analysis, we found that male patients are roughly 20% more vulnerable to the elevation in PSO risk by concomitant BD. This may that the latter exerts an overriding influence on PSO, since it is well established that PSO does not favour either sex in terms of incidence^33^, and likewise, BD is known to be gender-neutral or even show a modest degree of male predominance^34^.

Psoriasis is a systemic inflammatory condition, and among the most frequently cited comorbidities are obesity and other “lifestyle” diseases. In the case of BD, the association with metabolic status is less straightforward. A scarce number of reports indicate obesity is not common among BD patients^35,36^, but simultaneously, there are evidences to suggest that BD does render the patients more vulnerable to other features of metabolic syndrome^37,38^ (hypertension, diabetes, and dyslipidemia etc.). Harpsøe et al. found that with the exception of celiac disease (CD) and Raynaud’s phenomenon, BMI is associated with several autoimmune diseases^39^. Our results regarding the BMI covariate was rather interesting, as not only was there a positive correlation between BMI and the risk of PSO in BD patient with BMI over 23, but also a threefold-increase in the risk with sub-optimal BMI (under 18.5 kg/m^2^).

PsA is a chronic inflammation of both joints and soft connective tissues, that can manifest as either one or any combination of peripheral arthritis, spondylo-arthritis, and enthesitis/dactylitis. It is typically a sero-negative arthritis that begins as an oligo-arthritic disease but it often progresses to involve more joints. It is estimated that up to one third of PSO patients eventually develop PsA as well, and early recognition and prompt intervention is warranted because the inflammation tends to be severe with longer duration of the skin disease. Similarly, the BD-associated arthritis is also a nonerosive, sero-negative oligoarthritic disease which “migrates” to affect many other joints in the body. The prevalence of PsA in our PSO cohort was about 21% (3664 of 17,362), which was in line with published results from other studies. We found that BD individuals were twice more vulnerable to PsA as well, and other subgroup analyses revealed similar patterns that were seen in PSO, with the exception of female dominance.

The present investigation sets itself apart from earlier studies on the potential interrelationship between BD and PSO in several ways. To ensure the validity of the diagnosis, the authors limited data selection to those cases that were diagnosed and treated by certified dermatologists and rheumatologists only. Moreover, this study utilised most exhaustive healthcare database in Korea currently in operation, which contains a comprehensive record pertaining to BD and PSO/PsA during the span of 14 years (from 2002 to 2015). Furthermore, to shield the study results from as many confounding variables as possible, the outcomes values were not only adjusted for sex and age, but by gross income level (brackets) and disability status as well. Indeed, it has been suggested by a multitude of authors that chronic and multisystemic disorders, especially immune-mediated ones, may be influenced by socioeconomic factors and functional impairment^40^.

This is not to say that our study was completely immune from any shortcomings or limitations. Because the NHIS database was originally intended for insurance-related transactions, retrieval of data pertinent to disease severity or clinical subtypes of BD and PSO was not practical. In addition, the NHIS data, albeit incredibly voluminous, are *still* hospital-based records, and therefore the study may have been subject to selection bias. Finally, the sample size for the PsA cohort was rather small (*n* = 21) in comparison to the PsO counterpart. This inherent drawback has a lot to do with our rigorous selection criteria, but it affirms the long-held suspicion that PsA is being underdiagnosed^41^ (the prevalence is believed to be around 10–30%). The authors believe this to be significant because long-standing PsA can lead to irreversible joint destruction, and therefore early clinical suspicion and recognition is vital, as mentioned previously.

In summing up, the current investigation has backed up the long-held speculation about a potential immunological link between BD and PSO with a solid, population-based evidence. It was revealed that individuals with Behçet disease are twice more likely to develop psoriasis, and also psoriatic arthritis. The risk of psoriasis was correlated with BMI and was significantly higher in sub-optimal BMI. This added risk was pronounced in the male cohort, and tended to impact senile population, and this phenomenon may be related with the relatively poor prognosis of BD in males and PSO in older patients. Further studies are warranted to elucidate the mechanism behind this disease interaction and apply the insight for better-guided clinical practice.

## Methods

### Database (DB)

This study has been carried out in strict adherence to the Declaration of Helsinki. We used KNHIS-NSC data (NHIS-2016-2-162) provided by National Health Insurance Service (NHIS). The study was approved by the Institutional Review Board of *Hallym* Medical University *Chuncheon* Sacred Hospital (IRB No. 2016-05-052). The need for written informed consent was waived by the IRB, because the KNHIS-NSC data set consisted of deidentified secondary data for research purposes.

Regardless of her or his own volition, every South Korean national is covered by the NHIS, a comprehensive, state-enforced healthcare program that has been in effect since 1989. Eligible citizens are covered primarily through *community-based* plan or alternatively, *employee-based* plan. The data source for the current investigation was the national health information DB, which is the main body of NHIS-run servers that are considered well-suited for big-data research. The DB is the most comprehensive reservoir that harbours some 1.3 trillion cases of clinical/hospital administration records, including routine medical check-up results, prescription/treatment details, insurance transactions, and nationwide cancer/rare disease registries, from the years 2002–2015.

### Cohort definition

We extracted BD and psoriasis cohorts from the research DB using an algorithm suggested by the NHIS. The following two criteria had to be met simultaneously for BD cohort: those who had had at least two consecutive outpatient visits under the KCD7 (Korean Standard Classification of Diseases, 7th revision) Diagnosis Code of *M35.2* within the span of at least six months, *and* 2) those who had undergone pathergy test (prescription code E7113B). On the other hand, the PSO cohort was defined as those who had had at least two consecutive outpatient visits under one of the following KCD Diagnosis codes within the span of at least six months: *L40* (Psoriasis), *L40.00* (Psoriasis vulgaris), *L40.00* (Severe psoriasis vulgaris), *L40.08* (Other and unspecified psoriasis vulgaris), *L40.8* (Other psoriasis), and *L40.9* (Psoriasis, unspecified), *and* those who have been prescribed (a) topical calcipotriol/betamethasone dipropionate cream (Daivobet®) or gel formulation (Xamiol®), or calcipotriol solution (Daivonex®) for at least three consecutive months, *or* (b) those who have undergone ultraviolet (UV) phototherapy/photochemotherapy (prescription codes MM331, MM332, MM333, MM334, MM341, MM342, MM343, and MM344) or excimer laser for at least three consecutive months, *or* 3) those who have been prescribed methotrexate tablets (prescription code A04503121), or cyclosporin capsules (A01206211), or acitretin soft capsules (8806433047801 or 8806433047818) for at least three consecutive months. Similarly, the PsA cohort was obtained with the criteria of (1) those who had had at least two consecutive outpatient visits under one of the following KCD Diagnosis codes, with each visit being at least 6 months apart: M07* (Psoriatic arthropathies), *M07.0* (Distal interphalangeal psoriatic arthropathy), *M07.2* (Psoriatic spondylitis), and *M07.3* (Other psoriatic arthropathies) during at least two outpatient visits, *and either* (2) evidence of rheumatoid factor (RF) antibody testing or HLA-B27 typing *or* (3) those who had been prescribed disease-modifying anti-rheumatic drugs (DMARDs), or biologics/biosimilars approved for psoriatic arthritis, including cyclosporin (L04AD01), hydroxychloroquine (P01BA02), cyclophosphamide (L01AA01), methotrexate (L04AX03), mycophenolate (L04AA06), sulfasalazine (A07EC01), abatacept (L04AA24), rituximab (L01XC02), tocilizumab (L04AC07), adalimumab (L04AB04), certolizumab (L04AB05), etanercept (L04AB01), golimumab (L04AB06), and infliximab (L04AB02).

### Statistical analysis

Summary of demographic and baseline characteristics was constructed using descriptive analysis; the mean, maximum, minimum and standard deviation (S.D.) for quantitative variables and the frequency and percentage (%) for qualitative variables. Statistical relationships between BD and PSO/PsA were analysed using *χ*^2^ tests and binary logistic regression model. From the cohorts as defined above, odds ratios (OR), 95% confidence intervals (CI), and *p* values were calculated. The crude OR values were then adjusted by sex, age and gross income level (adjusted OR, or aOR). KSG, a one of the co-authors and an incumbent professor in medical statistics, oversaw the entire procedure of data analytics in the study. All statistical analyses were performed using SAS Enterprise Guide 6.1 M1 (SAS Institute Inc., Cary, NC, United States) and IBM SPSS software package for Windows (version 19.0, Chicago, IL, United States). All tests were two-sided and *p* values less than 0.05 were deemed statistically significant.

## Data Availability

The datasets presented in the current study are freely available from the corresponding. authors upon request.
